# Treatment of Breast Cancer With Gonadotropin-Releasing Hormone Analogs

**DOI:** 10.3389/fonc.2019.00943

**Published:** 2019-10-01

**Authors:** Maira Huerta-Reyes, Guadalupe Maya-Núñez, Marco Allán Pérez-Solis, Eunice López-Muñoz, Nancy Guillén, Jean-Christophe Olivo-Marin, Arturo Aguilar-Rojas

**Affiliations:** ^1^Unidad de Investigación Médica en Enfermedades Nefrológicas, Centro Médico Nacional Siglo XXI (CMN-SXXI), Instituto Mexicano del Seguro Social (IMSS), Hospital de Especialidades, Mexico City, Mexico; ^2^Unidad de Investigación Médica en Medicina Reproductiva, IMSS, Unidad Médica de Alta Especialidad No. 4, Mexico City, Mexico; ^3^Centre National de la Recherche Scientifique, CNRS-ERL9195, Paris, France; ^4^Unité d'Analyse d'Images Biologiques, Institut Pasteur, Paris, France; ^5^Centre National de la Recherche Scientifique, CNRS-UMR3691, Paris, France

**Keywords:** gonadotropin-releasing hormone (GnRH), gonadotropin-releasing hormone receptor (GnRHR), breast cancer, breast cancer adjuvant therapy, GnRH analogs, GnRH agonist, GnRH antagonist

## Abstract

Although significant progress has been made in the implementation of new breast cancer treatments over the last three decades, this neoplasm annually continues to show high worldwide rates of morbidity and mortality. In consequence, the search for novel therapies with greater effectiveness and specificity has not come to a stop. Among the alternative therapeutic targets, the human gonadotropin-releasing hormone type I and type II (hGnRH-I and hGnRH–II, respectively) and its receptor, the human gonadotropin-releasing hormone receptor type I (hGnRHR-I), have shown to be powerful therapeutic targets to decrease the adverse effects of this disease. In the present review, we describe how the administration of GnRH analogs is able to reduce circulating concentrations of estrogen in premenopausal women through their action on the hypothalamus–pituitary–ovarian axis, consequently reducing the growth of breast tumors and disease recurrence. Also, it has been mentioned that, regardless of the suppression of synthesis and secretion of ovarian steroids, GnRH agonists exert direct anticancer action, such as the reduction of tumor growth and cell invasion. In addition, we discuss the effects on breast cancer of the hGnRH-I and hGnRH-II agonist and antagonist, non-peptide GnRH antagonists, and cytotoxic analogs of GnRH and their implication as novel adjuvant therapies as antitumor agents for reducing the adverse effects of breast cancer. In conclusion, we suggest that the hGnRH/hGnRHR system is a promising target for pharmaceutical development in the treatment of breast cancer, especially for the treatment of advanced states of this disease.

## Introduction

Breast cancer (BC) is the most common cancer in women around the world. This disease represents 33% of all female cancer cases and it is responsible for ~15% of all cancer deaths among women worldwide. The high incidence rate of this disease could be associated with several factors, such as age, gender, reproductive factors, weight, personal and family history, lifestyle conditions, infectious agents, tobacco and alcohol consumption, obesity, and diet, among others.

According to the World Health Organization (WHO), it is estimated that, by 2025, there will be more than 20 million new cases of BC around the world (1). Additionally, in the majority of countries, BC is one of the main causes of death in women (2), and unfortunately, survival rates after 5 years of treatment are much worse for less developed countries, such as Gambia, Algeria, India, and Brazil (12–58.4%) in comparison with developed countries such as Japan, Sweden, and the United States (81.6–83.9%) (1, 3).

BC is a complex disease with considerable variations in clinical development, morphological aspect, and gene expression patterns. Traditionally, clinical staging is based on extension of the primary tumor, regional lymph nodes, and distant metastases. In addition, clinical staging includes histopathological assessments that comprise histological type, tumor grade or proliferation status, and lymphovascular invasion, and these are used as prognostic variables that reflect tumor biology and guide therapeutic management. Likewise, expression of estrogen receptor (ER) and progesterone receptor (PR) and the overexpression and/or amplification of the human epidermal growth factor receptor 2 (HER2) have been included in routine clinical practice worldwide to predict the prognosis of and the response to endocrine or humanized monoclonal antibody therapy (4, 5). Additionally, the molecular classification of BC is based on the expression levels of ER, PR, and HER2, allowing the stratification of this disease into three major subtypes: luminal, HER2 overexpression, and triple-negative breast cancer (TNBC) tumors (6). Luminal is the most common tumor subtype, characterized by the expression of sex-steroid receptors (ER and PR). Recently, the expression levels of Ki67, a cellular marker of proliferation, have been employed to discriminate luminal A and luminal B subtypes (4). In general, luminal tumors respond well to hormone therapy but poorly to conventional chemotherapy. HER2-overexpressing tumors are characterized by the phenotype: ER negative (ER–), PR negative (PR–), and HER2 positive (HER2+). Although these types of tumors are associated with aggressive disease and decreased survival, the chemotherapy treatment in combination with novel anticancer agents, including monoclonal antibodies and small tyrosine kinase inhibitors, result in an improvement in disease-free survival (7). TNBC refers to a group of tumors that do not express either hormone receptors (ER–, PR–) or HER2 receptor (HER2–). This tumor type is of particular clinical interest because of its aggressive course, due to the deficient expression of potential therapeutic targets; thus, chemotherapy is the only option for these patients. Compared with the other subtypes, TNBC tumors are associated with younger patients, their frequency is more prevalent in African-American populations, and they are clinically more aggressive (8). Although TNBCs are sensitive to chemotherapy, response rates are low and, in addition, the prognosis remains poor. In patients with TNBC, recurrence occurs earlier, and the majority of the deaths occur within the first 5 years after diagnosis, underscoring the importance of identifying specific therapeutic targets for this cancer subtype.

Despite that the use of genomic/gene expression arrays allows the incorporation of prognostic or predictive markers that provide additional information for conventional clinical staging (9), the development of targeted drugs for BC treatment remains in evolution. Therefore, the characterization of specific tumor receptors or overexpressed receptors and the development of novel therapies based on new peptide hormone analogs as targets for cancer therapy have been proposed. Among these targets, the human gonadotropin-releasing hormone type I (hGnRH-I), also called the hypothalamic luteinizing hormone-releasing hormone (LHRH), and its receptor, the human gonadotropin-releasing hormone receptor type I (hGnRHR-I), also known as the hypothalamic luteinizing hormone-releasing hormone receptor (LHRH-R), have been predicted as potentially therapeutic agents for their antitumor activities (10–13).

## Functions of hGnRH-I, hGnRH-II, and hGnRHR-I in Reproduction

**Figure 1:**
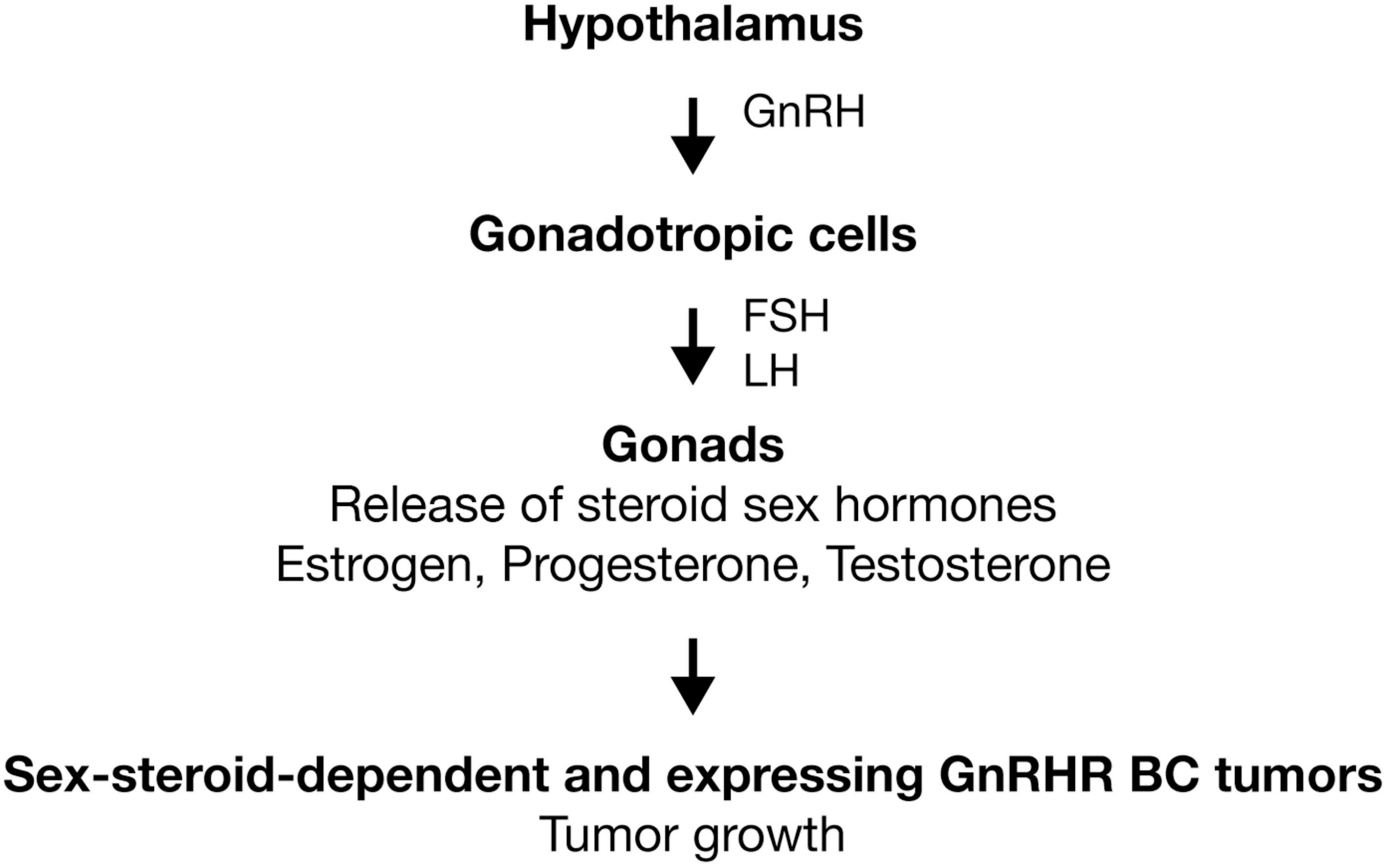
Activation of GnRHR in gonadotropic cells. GnRH is produced in hypothalamus and released in a pulsatile fashion to act primarily on the anterior pituitary. Here, the GnRHR is expressed in the membrane of gonadotropic cells (gonadotropes). Receptor activation stimulates the synthesis and secretion of LH and FSH. In gonads, gonadotropins trigger gametogenesis as well as the synthesis and release of steroid sex hormones (estrogen, progesterone, and testosterone). In sex-steroid-dependent BC tumors, sexual hormones promote the tumoral growth. In BC tumors that express GnRHR, analogs of GnRH could improve the treatments anticancer by the inhibition of tumoral growth. Activation (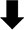).

GnRH-I is a hypothalamic hormone first isolated from porcine hypothalamus (14). On the other hand, its receptor was purified and characterized first from bovine anterior pituitaries (15). At present, it is well-established that both molecules comprise the signaling complex that controls sperm and ovary maturation, as well as steroidogenesis in gonads (16–18). Human GnRH-I (hGnRH-I) is a decapeptide hormone described for first time in 1971 by the group of Schally (Tables 1, 2) (19). Seeburg and Adelman cloned the encoding gene for the first time in 1984 (76). In humans, the *GnRHI* gene is located on chromosome 8p11.2-p21 and is composed of four exons separated by three introns (77). This hormone is produced in the hypothalamus by GnRH neurons for release in a pulsatile fashion into the hypophyseal portal circulation to act primarily on the anterior pituitary, where it binds its receptor, the hGnRHR-I, in gonadotropic cells to stimulate the synthesis and secretion of pituitary gonadotropic hormones: luteinizing hormone (LH) and follicle-stimulating hormone (FSH) (Figure 1) (18). In the gonads, gonadotropins trigger gametogenesis as well as the synthesis and release of steroid sex hormones in females and males (Figure 1). Likewise, gonadal steroids are able to regulate hGnRH-I secretion through both positive and negative feedback (78). hGnRH-I is synthesized as a prohormone. The full sequence is a 92-amino-acid (aa) peptide in which the first 23 aa are a signal peptide followed by the functional GnRH decapeptide. Later, an amidation/proteolytic processing signal (Gly-Lys-Arg) is found, and finally, there is a 56-aa region known as the GnRH-associated peptide (GAP), which is co-secreted with GnRH and which appears to be involved in the processing and packaging of the decapeptide (79, 80). In humans, there is a second subtype of GnRH denominated hGnRH-II (Tables 1, 2). It is also a decapeptide hormone that differs from hGnRH-I in three amino acids (GnRH-II; His^5^, Trp^7^, Tyr^8^-GnRH-I) and that is encoded by the gene *GnRH2*, which has been mapped in chromosome 20p13 (58). hGnRH-II is found in the midbrain region and serves principally as a neurotransmitter and stimulator of sexual behavior (81). However, it has been shown that hGnRH-II can stimulate gonadotropin release *in vivo* through its binding to hGnRHR-I (82). The expression of hGnRH-I and hGnRH-II is differential. By hGnRH-I, its expression is higher in the brain (18). In the opposite site, hGnRH-II is ubiquitously expressed in different systems, such as thoracic (heart, lung, and aorta), digestive (salivary gland, stomach, and intestine), endocrine (adrenal, pancreas, and thyroid), and immune (tonsil, leukocyte, and lymph node) (83, 84).

**Table 1:** Chemical structure of hGnRH-I agonists (GnRHa) and hGnRH-II agonists (GnRHa-II) evaluated against breast cancer.

	**Sequence**	**Main clinical indication (drugbank and FDA)**	**Clinical use in breast cancer**
hGnRH-I (Gonadorelin)	Pyro-Glu^1^-His^2^-Trp^3^-Ser^4^-Tyr^5^- Gly^6^-Leu^7^-Arg^8^-Pro^9^-Gly^10^-NH_2_ (19).	For evaluating the functional capacity and response of the gonadotropes of the anterior pituitary.	Information not available, only for GnRHa.
		For evaluating residual gonadotropic function of the pituitary following removal of a pituitary tumor by surgery and/or irradiation.	
		Ovulation induction therapy.	
**GnRHa**			
Triptorelin	Pyro-Glu^1^-His^2^-Trp^3^-Ser^4^-Tyr^5^-**D-Trp**^6^-Leu^7^-Arg^8^-Pro^9^-Gly^10^-NH_2_ (20–24).	Palliative treatment of advanced prostate cancer.	In premenopausal women with early BC letrozole in combination with triptorelin induces a more intense estrogen suppression than tamoxifen with triptorelin (25).
			In healthy premenopausal women coadministration of triptorelin and exemestane resulted in greater estrogen suppression than when triptorelin was given alone (26).
			In premenopausal women with HR+ early BC, adjuvant treatment with exemestane plus ovarian suppression, as compared with tamoxifen plus ovarian suppression, significantly reduced recurrence (24).
		Controlled ovarian hyperstimulation therapy.	In premenopausal women with BC, concurrent administration of triptorelin and chemotherapy, compared with chemotherapy alone, was associated with higher long-term probability of ovarian function recovery, however there was no significant difference in DFS (27).
			In premenopausal women with BC, treatment with exemestane plus triptorelin had estradiol levels consistent with levels reported in postmenopausal women on aromatase inhibitors (23).
			In premenopausal women who received adjuvant chemotherapy for HR+, HER2 negative (HER2-) BC, neither detrimental, nor beneficial effect of concurrent administration OFS was detected (28).
			In premenopausal women with stage cT2 to 4b, any N, M0, HR+, and HER2- BC receiving letrozole neoadjuvant, OFS was achieved more quickly and maintained more effectively with degarelix than with triptorelin (29).
			In premenopausal women with early BC undergoing OFS with triptorelin, the treatment with letrozole and zolendronic acid, improves DFS (30).
Goserelin	Pyro-Glu^1^-His^2^-Trp^3^-Ser^4^-Tyr^5^ **D-Ser(But)**^6^-Leu^7^-Arg^8^-Pro^9^-**Aza-Gly**^10^-NH_2_ (31–40).	In combination with flutamide for management of locally confined carcinoma prostate.	In pre y perimenopausal women with metastatic BC, goserelin produced objective response rates and duration of remission at least comparable to those seen following oophorectomy (41).
		Palliative treatment of advanced carcinoma prostate.	In premenopausal women with early BC, the addition of goserelin to ajuvant chemotherapy was associated with more benefit in DFS and overall survival rates (42).
		The management of endometriosis.	In premenopausal women with HR+ BC, OFS with goserelin plus tamoxifen compared with tamoxifen only provided more benefit in DFS (43).
			In premenopausal women with prior endocrine-resistant HR+, HER2- advanced BC, palbociclib combined with fulvestrant, and goserelin was an effective treatment to extend DFS (44).
		Use as an endometrial-thinning agent prior to endometrial ablation for dysfunctional uterine bleeding.	In premenopausal women at ≥30% lifetime risk breast cancer, OFS with goserelin is a potential regimen for BC risk reduction (45).
			In premenopausal women with HR+, HER2-, tamoxifen-pretreated metastatic BC, fulvestrant plus goserelin provides a new option for the treatment (46).
		Palliative treatment of advanced BC in pre- and perimenopausal women.	In premenopausal o perimenopausal women with advanced HR+, HER2- BC, overall survival was longer with a CDK4/6 inhibitor plus endocrine therapy (including goserelin) than endocrine therapy alone (47, 48).
Buserelin	Pyro-Glu^1^-His^2^-Trp^3^-Ser^4^-Tyr^5^-D-**Ser(But)**^6^-Leu^7^-Arg^8^-**Pro-NHET**^9^ (49–53).	May be used in the treatment of HR+ cancers such as prostate cancer o BC.	In premenopausal women with metastatic BC, buserelin was associated with objective remission and stable disease (54, 55).
		May be used in estrogen-dependent conditions (such as endometriosis or uterine fibroids).	In premenopausal women with BC, buserelin plus cytostatics more effectively caused ovarian ablation than cytostatic treatment alone (56).
		May be used in assisted reproduction.	In premenopausal women with advanced BC, the effect of cyclophosphamide, doxorubicin and fluoruracil plus buserelin showed a high response rate (57).
			In premenopausal women with BC, combining OFS with buserelian and tamoxifen was superior to treatment with buserelin or tamoxifen alone by objective response rate, more DFS and longer overall survival (51).
hGnRH-II	Pyro-Glu^1^-His^2^-Trp^3^-Ser^4^-His^5^- Gly^6^-Trp^7^-Tyr^8^-Pro^9^-Gly^10^-NH_2_ (58).	Information not available.	Information not available.
		EXAMPLES OF USES REPORTED IN CANCER MODELS hGnRH-II may be involved in the inhibition of endometrial cancer cell growth (HEC-1A) (59). hGnRH-II can promote apoptosis rate and inhibit cell proliferation of estrogen receptor-negative endometrial cancer cells (HEC-1A) in a dose-dependent manner (60).	
**GnRHa-II**			
[D-Lys6]-GnRH-II	Pyro-Glu^1^-His^2^-Trp^3^-Ser^4^-His^5^-D-Lys ^6^-Trp^7^-Tyr^8^-Pro^9^-Gly^10^-NH_2_ (21).	Information not available.	Information not available.
		EXAMPLES OF USES REPORTED IN CANCER MODELS [D-Lys6]-GnRH-II has potent antiproliferative effect on SKOV-3 human ovarian cancer cell line (61). Cytotoxic conjugate prepared by the attachment of the chemotherapeutical agent daunorubicin to [D-Lys6]-GnRH-II had significantly higher long-term cytotoxic than cytostatic effects in human breast (MCF-7) and colon (HT-29) cancer cell lines (62).	

*Pyro-Glu; pyroglutamic acid. His; L-histidine. Trp; L-tryptophan. Ser; L-serine. Tyr; L-tyrosine. Gly; L-glycine. Leu; L-leucine. Arg; L-arginine. Pro; L-proline. D-Trp; D-tryptophan. D-Ser(But); D-serine ter-butyl. NHET; N-ethylamide. Aza-Gly; azaglycine (stands for glycine in which the α-CH has been replaced by a nitrogen atom); BC, breast cancer; HR+, hormone receptor positive; DFS, disease free survival; HER2-, HER2 negative; OFS, ovarian function suppression; FDA, United States Food and Drug Administration (https://www.accessdata.fda.gov/scripts/cder/daf/); DRUGBANK: https://www.drugbank.ca*.

**Table 2:** Chemical structure of hGnRH-I antagonists (GnRH-ant) and hGnRH-II antagonists (GnRH-ant-II) evaluated against breast cancer.

	**Sequence**	**Main clinical indication (drugbank and FDA)**	**Clinical use in breast cancer**
hGnRH-I (Gonadorelin)	Pyro-Glu^1^-His^2^-Trp^3^-Ser^4^-Tyr^5^-Gly^6^-Leu^7^-Arg^8^-Pro^9^-Gly^10^-NH_2_ (19).	For evaluating the functional capacity and response of the gonadotropes of the anterior pituitary.	Information not available.
		For evaluating residual gonadotropic function of the pituitary following removal of a pituitary tumor by surgery and/or irradiation.	
		Ovulation induction therapy.	
**GnRH-ant**			
Cetrorelix	**Ac-D-2Nal**^1^-**D-Phe(4-Cl)**^2^-**D-3Pal**^3^-Ser^4^-Tyr^5^-D-Cit^6^-Leu^7^-Arg^8^-Pro^9^-**D**-**Ala**^10^-NH2 (63, 64).	For the inhibition of premature LH surges in women undergoing controlled ovarian stimulation.	Information not available.
		EXAMPLES OF USES REPORTED IN CANCER MODELS In N-nitrosobis (2-oxopropyl) amine induced ductal pancreatic cancer in female Syryan golden hamsters, cetrorelix with somatostatin analogs resulted in market reduction of tumor pancreas weight and increased of apoptosis (65). In rats bearing Dunning R3327H transplantable prostate carcinoma, therapy with microgranules of BF-75 significantly decreased tumor growth (66). Cetrorelix directly inhibit growth of mammary tumor cells [MCF-7 and MCF7 MIII (64, 67)]. In dimethylbenzathracene induced mammary carcinoma in female Sprague Dawley rats, cetrorelix was effective in reducing tumor mass (68). Cetrorelix has significantly stronger antiproliferative effects on human endometrial (HEC-1A and Ishikawa) and ovarian cancer (EFO-21, OVCAR-3, SK-OV-3) cells than Triptorelin (69). In patients with ovarian or mullerian carcinoma resistant to platinum chemotherapy treated with cetrorelix, partial remission and disease stabilization was observed (70). In mouse pheochromocytoma cells and mouse tumor tissue-derived cell line, cetrorelix showed significant anti-tumor effects, leading to reduction in cell viability (71).	
hGnRH-II	Pyro-Glu^1^-His^2^-Trp^3^-Ser^4^-His^5^-Gly^6^-Trp^7^-Tyr^8^-Pro^9^-Gly^10^-NH_2_ (58).	Information not available.	Information not available.
**GnRH-ant-II**			
Triptorelix-1	**Ac-D2Nal**^1^-**D-4Cpa**^2^-**D-3Pal**^3^-Ser^4^-**Tyr**^5^-**D-Cit**^6^-Trp^7^-Tyr^8^-Pro^9^-**D-Ala**^10^-NH2 (72).	Information not available.	Information not available.
		EXAMPLES OF USES REPORTED IN CANCER MODELS Triptorelix induced growth inhibition of PC3 prostate cancer cells *in vitro* and inhibited growth of PC3 cells xenografted into nude mice (72, 73).	
SN09-2	**Ac-D2Nal**^1^-**D-Phe(4-Cl)**^2^-**D-3Pal**^3^-Ser^4^-**Phe**^5^-**D-Lys**^6^-Trp^7^-Tyr^8^-**Arg**^9^-**D-Ala**^10^-NH2 (72).	SN09-2 reduced the growth and increased apoptosis of PC3 prostate cancer cells and was associated with decreased membrane potential and mitochondrial dysfunction (72).	
[Ac-D2Nal1, D-4Cpa2, D-3Pal3,6, Leu8, D-Ala10]-GnRH-II	**Ac-D2Nal**^1^**-D-4Cpa**^2^- **D-3Pal**^3^-Ser^4^-His^5^**-D-3Pal**^6^-Trp^7^-**Leu**^8^-Pro^9^**-D-Ala**^10^-NH2 (74, 75).	[Ac-D2Nal1, D-4Cpa2, D-3Pal3,6, Leu8, D-Ala10]-GnRH-II induce apoptosis in human endometrial (HEC-1A, HEC-1B and Ishikawa), ovarian (OVCAR-3 and EFO-21) and breast cancer cells (MCF-7 and T47-D) (74, 75).	

*Pyro-Glu; pyroglutamic acid. His; L-histidine. Trp; L-tryptophan. Ser; L-serine. Tyr; L-tyrosine. Gly; L-glycine. Leu; L-leucine. Arg; L-arginine. Pro; L-proline. Ac; acetyl group. D-Nal; D-naphthyIalanyl. D-Phe(4-Cl) or D-4CPa; 4-Chloro-D-phenylalanine. D-3Pal; 3-D-pyridylalanine. D-Cit; D-citrulline. D-Lys; D-lysine. D-Ala; D-alanine. Underlined amino acids; L-amino acids changed with respect to the parent peptide; FDA: United States Food and Drug Administration (https://www.accessdata.fda.gov/scripts/cder/daf/); DRUGBANK: https://www.drugbank.ca*.

hGnRHR-I is a member of the superfamily of G protein-coupled receptor (GPCR) and, according to its aa sequence, this receptor belongs to the family of Rhodopsin. The structure of hGnRHR-I is similar to that of other GPCRs; it is a single amino-acid chain that shows an extracellular amino-terminal, seven transmembrane domains, and three extracellular and three intracellular loops (17). In the case of hGnRHR-I, this was cloned and characterized from human pituitary for the first time in 1992 (85). In comparison with other GPCRs, there are specific characteristics that distinguish the human GnRHR receptor, including the lack of a cytoplasmic carboxyl-terminal tail. This characteristic has been associated with inefficient desensitization on hGnRH-I stimulation and relatively slow internalization (86–88). Another feature of hGnRHR-I is the presence of a lysine at position 191. Research works have demonstrated the presence of this residue in human receptors and it is possibly implicated in reducing hGnRHR expression on the cellular surface (89, 90).

A second type of GnRHR, termed GnRHR-II, occurs in mammals. The search for this gene in human genome database revealed its presence as a putative gene (*GnRHR2*) on chromosome 1q12 (91, 92). Although the sequences display 40% of identity with hGnRHR-I (93), analysis of the corresponding reading frame shows the presence of a premature stop codon (94, 95). Nonetheless, the expression of mRNA and several alternatively spliced transcripts derived from GnRHR2 were demonstrated; in all of these, the premature stop codon was retained (91, 92). In conclusion, all of these observations support the notion that this gene is not functional and there is no GnRHR type II in humans.

## Physiology of GnRH-I, GnRH-II, and hGnRHR-I in BC

The presence of hGnRH-I, hGnRH-II, and hGnRHR-I has been demonstrated in tumors of the reproductive tract, such as ovarian cancer (96–98), prostate cancer (99–101), and BC (102–104).

Numerous studies have shown the expression of hGnRH-I, hGnRH-II, and hGnRHR-I in BC tissue and provide a rationale for their use as a molecular target for the treatment of BC. hGnRH-I binding sites were reported in the biopsies of primary human carcinoma tissues and were localized in the cytoplasm of 64% of cases of invasive ductal carcinoma (102, 105). At the same time, nearly 50% of BC specimens possess hGnRH-I binding sites on their surfaces (106, 107). In TNBC tissues, GnRHR-I mRNA was detected in up to 70% (20, 107–110). Finally, in different human BC cell lines, including TNBC cell lines, either hGnRH-I-binding sites or hGnRH-II-binding sites were reported (20, 21, 63, 75, 107, 109, 111).

## Effects of hGnRH-I, hGnRH-II, and hGnRHR-I on Cell Proliferation and Cell Migration in BC

The hGnRHR-I, hGnRH-I, and hGnRH-II systems have shown specific characteristics in cancer cells. For example, although the transcripts expressed in malignant tumor share coding region and protein from the pituitary, their expression levels are lower in comparison with those on gonadotropic cells (112, 113). In tumor cells, there are high-affinity binding sites or low-affinity binding sites by hGnRH-I and hGnRH-II; the dichotomy of the GnRH agonist and antagonist is not clearly present and the mechanisms activated by this receptor are different from those of the signaling pathways present in the pituitary–gonadal axis (96, 97, 102, 114–118). All of these aspects should be studied in greater depth in order to achieve the clinical implementation of hGnRHR-I, hGnRH-I, and hGnRH-II as therapeutic targets in human extra-pituitary tissue.

The antitumoral functions of hGnRH-I or GnRH-II and the hGnRHR-I in BC have been associated with the reduction of cell proliferation, invasion, and migration (10, 119–124). The molecular mechanisms employed have been explored in several research models. The reduction of cell proliferation in malignant cells is achieved through coupling between this receptor and G_i_ protein, as well as by crosstalk between hGnRHR-I and growth factor receptors (125). In the MCF-7 cell line, the overexpression of hGnRHR-I by adenoviruses was able to decrease cellular proliferation (117). The inhibitory cell growth effect promoted by hGnRHR-I activation in this cell line was mediated by the indirect inactivation of both the epidermal growth factor (EGF) receptor and the insulin-like growth factor (IGF) receptor (126, 127). Furthermore, it was additionally shown that hGnRH-I analogs were also able to suppress the EGF-induced activation of mitogen-activated protein kinase (MAPK) and the dephosphorylation of EGF receptors via the activation of phosphotyrosine phosphatase (128, 129). In a mouse model treated with GnRH agonist, the expression of EGF and IGF receptors was decreased (130). In 4OH-Tamoxifen-resistant MCF-7 and T47D-TR cell lines, GnRH agonists were able to abolish cell proliferation, blocking EGF-receptor autophosphorylation and ERK1/2 activation and reducing EGF-induced c-fos protein expression (131, 132).

The cellular mechanism that explains how the hGnRH-I/hGnRHR-I system inhibits cell invasion has been studied in the MDA-MB-231 cell line. The activation of hGnRHR-I by GnRH agonist results in decreased expression of the Rho GTPase-Activating Protein 18 (ARHGAP18) and the concomitant increase in the activation of RhoA (49, 124). The overactivity of RhoA triggers intracellular signaling pathways to produce more stress fibers, more focal adhesions, and major substrate adhesion, which in turn inhibit cellular migration. On the other hand, in MDA-MB-231 cells and in mesenchymal-transformed MCF-7 cells (MCF-7-EMT), the administration of GnRH agonist was also able to block the expression and activity of S100 calcium binding protein A4 (S100A4) and cysteine-rich angiogenic inducer 61 (CYR61). Considering that both proteins play important roles in cellular invasion and metastasis, their inhibition by GnRH analogs could be associated with the decrease of invasion observed (133).

In the case of hGnRH-II analogs, these exert an effect on the reduction of tumor growth rates by means of the induction of apoptosis, demonstrating a novel hGnRH-II signaling pathway that is active in tumors (69, 74, 75, 118). The molecular mechanism related with this antitumor effect mediated by the apoptotic pathway results in the activation of stress-induced MAPK or Bax via p38 and JNK. The deficiency of mitochondrial membrane potential and apoptotic cell death via the dose-dependent activation of caspase-3 was demonstrated by the use of several hGnRH-II analogs (69, 74, 75, 118). On the other hand, the ability to reduce metastases in BCs has also been exhibited by GnRH-II agonists (63).

All of the previously mentioned information supports the use of hGnRHR-I as a clinical asset against BC. Herein, in this review, we explored recent information on the development and use of GnRH analogs, such as agonists (defined in this work as GnRHa by hGnRH-I agonists and GnRHa-II by hGnRH-II agonists), antagonists (defined in this work as GnRH-ant by hGnRH-I antagonists and GnRH-ant-II by hGnRH-II antagonists), non-peptide GnRH antagonists, and cytotoxic analogs of GnRH and their possible implications as antitumor agents against BC.

## Analogs of GnRH Used Against BC

After the discovery of the amino-acid sequence of hGnRH-I in 1971, the development of synthetic analogs began with the intent to synthesize stimulating and blocking variants. This process has been very intense due its clinical application, and as a consequence, ~5,000 analogs of this hormone were synthesized between 1972 and 2016 (134). In the case of stimulating variants or agonists, their design has been centered on improving receptor binding and subsequent activation. On the other hand, blocking variants or antagonists have been designed with strong receptor binding but without activation.

## GnRH-I Agonists

The clinical applications of GnRH agonists were limited by their short half-life (2–4 min) promoted by the rapid cleavage among the amino acids at positions 5–6, 6–7, and 9–10 (134, 135). For this reason, the development of long-acting agonists was based on two principal criteria: first, they were derived from native hGnRH-I by adding D-amino acids at position number 6, 9, or 10 instead of L-amino acids. With this change, the agonists were resistant to degradation and also increased their receptor binding sites (134, 135). Second, because an essential role of NH2 and the COOH termini is played in binding to the receptor, usually Pro^9^- and/or Gly^10^-NH2 are modified by Pro^9^-NHET or Pro^9^-Aza-Gly^10^-NH2 to increase half-life and receptor occupancy time (136). All of these modifications have improved the ability of these agonists to activate their receptor by several hundreds of magnitude in comparison with the native hGnRH-I.

**Figure 2:**
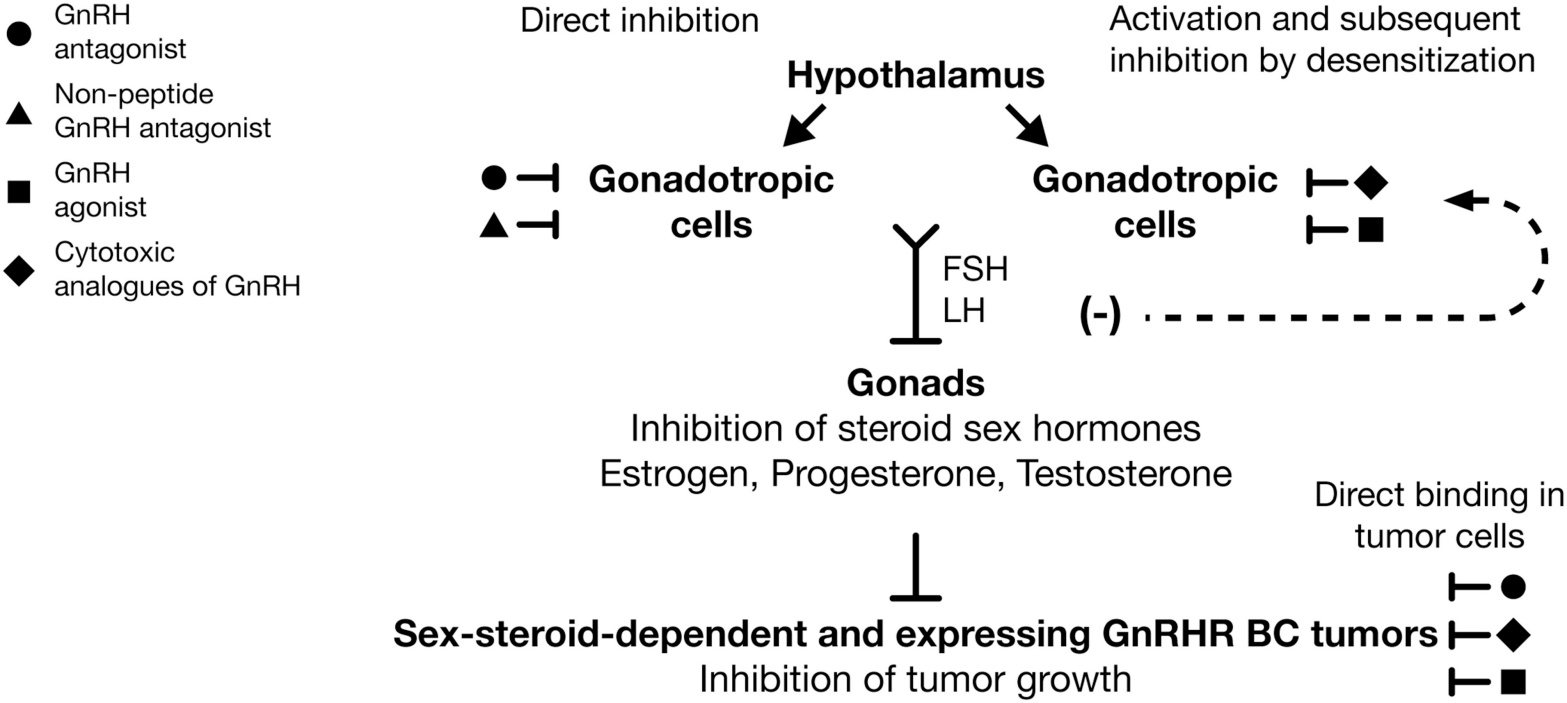
Use of GnRH analogs as adjuvant therapy against breast cancer. GnRH antagonists (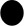) and non-peptide GnRH antagonists (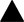) are able to reduce FSH and LH expression by direct inhibition of GnRHR in gonadotropic cells. GnRH agonists (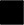) and cytotoxic analogs of GnRH (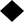) are able to reduce gonadotropin hormones levels after GnRHR desensitization in gonadotropic cells. Suppression of FSH and LH evoke diminution of estrogen, progesterone, and testosterone levels and the subsequent inhibition of growth in dependent sex-steroid tumors. Likewise, GnRH agonists (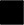), GnRH antagonists (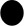), and cytotoxic analogs of GnRHR (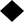) have direct antitumor effects over cancer cells, promoting in those the inhibition of cell growth. The systemic and local effect of GnRH analogs could improve the clinical response in BC patients, principally those that are treated with combined therapies. Inhibition (⊥), desensitization (**−**).

At present, it is clear that acute exposure to GnRHa produces adverse effects, such as hot flashes (“flares”) caused by the increase and sudden decrease of gonadotropin hormones and steroid hormones. This is due to the continuous administration of GnRHa producing early receptor activation and to its subsequent downregulation and desensitization in gonadotropic cells (Figure 2). These effects decrease the secretion of LH and FSH and the lack in sex-steroid hormones (estrogens, testosterone, and progesterone) with the concomitant appearance of “chemical castration” (Figure 2). This castration effect highlights the potential use of the treatment of early stage of endocrine-responsive tumors of BC, with GnRHa as adjuvant therapy in combination with Tamoxifen or aromatase inhibitors (Figure 2). Thus, it is important to bear in mind that the GnRH analogs employed in clinical practice commonly are administered in combination with estrogen antagonists or chemotherapy agents and not in direct form. Although this is primarily due to ethical reasons, it results in a limited understanding of the direct effects that GnRH analogs could exert on BC. Similarly, it is important to consider that, although the majority of the results obtained through studies carried out in animal models or in cell lines reflect or are in agreement with the effects observed in clinical studies, they must be interpreted in terms of their clinical application with caution and more studies are needed to generalize their use as therapeutic tools.

Administration of GnRHa as adjuvant therapy against BC is able to reduce, in a reversible manner, circulating concentrations of estrogen (ovarian suppression) in premenopausal women through its action on the hypothalamus–pituitary–ovarian axis (Figure 2). These effects reduce tumor growth that is sensitive to circulating estrogens either by preventing the development of distant metastases or by extending disease-free survival (DFS), and therefore overall survival (OS) (137, 138). The positive effect of GnRHa as adjuvant treatment in premenopausal women with ER+ tumors was shown by the analysis of 16 randomized trials. According to these data, when GnRHa was added to Tamoxifen or chemotherapy treatment or both, there was a reduction in the recurrence of BC and fewer people died after recurrence of the disease (139). On the other hand, the beneficial effect of GnRHa in TNBC was also shown. In this case, a meta-analysis of randomized controlled trials supports the use of GnRHa in the clinical setting as adjuvant therapy for the management of TNBC tumors (140).

Additionally, several research groups have demonstrated that GnRHa exerts direct anticancer activity on malignant tissue including mammary, which is independent from the suppression of the ovarian steroid synthesis and secretion (Figure 2) (50, 141). Based on the information mentioned previously, it is possible to propose that GnRHa exerts a systemic effect on breast tumors by ovarian suppression and a local effect by hGnRHR-I activation (Figure 2). Next, we describe the positive effects of specific GnRH-I and GnRH-II agonists employed as therapeutic agents against BC.

### Triptorelin

Triptorelin (Table 1) is a GnRHa that has been approved in the European Union as an adjuvant endocrine therapy in combination with Tamoxifen or AI to treat endocrine-responsive, early-stage BC (22). This management blocks the action of estrogen in ER+ BC cells (Tamoxifen) or inhibits the production of estrogen (AI, GnRHa) in patients (Figure 2) (23, 142). The benefits of Triptorelin as adjuvant therapy were investigated by randomized, open-label, phase III trials (22, 24). In these studies, 2,359 premenopausal women with hormone-receptor-positive early BC received treatment with Exemestane plus ovarian suppression with Triptorelin and 2,358 received Tamoxifen plus ovarian suppression, also with Triptorelin. After a median follow-up of 68 months of treatment, DFS at 5 years was 91.1% in the Exemestane-ovarian suppression group and 87.3% in the Tamoxifen-ovarian suppression group. The rate of being free from BC at 5 years was 92.8% in the Exemestane-ovarian suppression group, as compared with 88.8% in the Tamoxifen-ovarian suppression group. Overall survival did not differ significantly between the two groups. These data show the favorable effect that Triptorelin as ovarian suppressor has over the BC treatment.

The antitumoral effects of Triptorelin, Cetrorelix, and the GnRHa-II [D-Lys6]-GnRHII also have been evaluated in *in vitro* and *in vivo* research models (21). In MCF-7, HCC 70, and T47-D cell lines and, in a mouse model, a reduction in metastasis and significant inhibition of bone metastasis formation were observed (21). On the other hand, Triptorelin and [D-Lys(6)]-GnRHII were able to inhibit EGF-receptor signaling transductional pathway and restored sensitivity to 4-OH-Tamoxifen in 4OH-Tamoxifen-resistant MCF-7 cells and T47D-TR cells [81. In TNBC cell lines, MDA-MB-231 and HCC1806, the administration of Triptorelin either individually or in combination with chemotherapeutic agents such as Cisplatin, Docetaxel, and AEZS-112, and PI3K/AKT inhibitors (Perifosine, AEZS-129), ERK inhibitor (AEZS-134), and dual PI3K/ERK inhibitor AEZS-136 showed antiproliferation activity. In both cell lines, synergistic effects took place when Triptorelin was combined with Cisplatin. In HCC1806 cells, synergy occurred when Triptorelin was applied with PI3K/AKT inhibitors Perifosine and AEZS-129. In MDA-MB-231 cells, synergy was observed after co-treatment with Triptorelin and the ERK inhibitor AEZS-134 and dual PI3K/ERK inhibitor AEZS-136 (20).

### Goserelin

Goserelin (Table 1) is a GnRHa approved in 1989 by the Food and Drug Administration (FDA) for the treatment of BC as adjuvant endocrine therapy due to its abilities to reduce circulating concentrations of estrogen (Figure 2). One study demonstrated that, in premenopausal patients with ER-positive BC, Goserelin offers an equivalent and well-tolerated alternative to Cyclophosphamide, Methotrexate, and Fluorouracil (CMF) chemotherapy. In this case, Goserelin was also able to display equivalent DFS compared to cytotoxic chemotherapy, but with a more favorable safety profile (31, 32). Moreover, in a randomized trial performed in 2003 by the International Breast Cancer Study Group (IBCSG), it was shown that, in ER-negative patients, the course of the disease was improved if the patients received CMF in combination with Goserelin. Additionally, in women under the age of 39 years, the combination of chemotherapy followed by Goserelin resulted in superior DFS (33).

In a retrospective study performed in patients aged <40 years diagnosed with invasive BC, the administration of Goserelin as systemic adjuvant therapy following surgical removal of the primary tumor and a chemotherapy scheme with CMF demonstrated better results in comparison with the administration of CMF and Goserelin alone (34). In this study, the administration of Goserelin plus neoadjuvant chemotherapy exhibited higher rates of pathological Complete Response (pCR) and a markedly decreased proliferation index compared with the neochemotherapy-alone group, principally in ER- and/or in PR-negative tumors (34). Karlsson et al. reported similar results. These authors observed that the combination of CMF and Goserelin in premenopausal patients with ER-positive tumors showed a significant improvement of DFS and a BC-free interval in comparison with the administration of CMF and Goserelin alone (35). A positive effect on the patient's quality of life after the administration of Goserelin, in comparison with CMF chemotherapy alone, was demonstrated in premenopausal and perimenopausal patients with early BC (36). Finally, in a randomized study in patients with ER-negative BC, the administration of Goserelin during the course of chemotherapy protected ovarian function, reducing the risk of early menopause and improving prospects for fertility (37).

Beneficial effects due to the combination of Goserelin and AI or Goserelin and Tamoxifen also were revealed. In the former case, simultaneous administration of Goserelin and 4-hydroxyandrostenedione (4-OHA) improved the clinical response of patients with BC (38). Likewise, the combination of Goserelin and Tamoxifen in premenopausal patients with advanced BC increased progression-free survival time in comparison with the use of a GnRH agonist alone (39, 40).

### Buserelin

Buserelin is another hGnRHa (Table 1). The use of ^125^I-Buserelin in 235 samples of biopsies from pre- and postmenopausal women demonstrated the presence of high-affinity binding sites for GnRH (50).

On the other hand, several studies have shown the beneficial effects in the use of combined therapies of Tamoxifen and Buserelin or AI and Buserelin in patients. In 161 premenopausal women with metastatic BC, the combined treatment with Buserelin and Tamoxifen evoked a significant improvement in patient survival in contrast with treatment with either drug alone (51). Furthermore, a couple of studies performed with metastatic male BC patients demonstrated that the treatment with an AI (Letrozole) or antiandrogens (cyproterone acetate) in combination with GnRHa (Leuprolide, Triptorelin, Buserelin, and Goserelin) could be associated with greater survival in comparison with the monotherapy (52, 53).

Several groups have reported that hGnRHR-I activation by Buserelin in cell lines is also able to evoke the antitumor activities observed in malignant tissues. In MCF-7 cells, Buserelin promotes a potent antiproliferative effect (114, 143). On the other hand, in the TNBC cell line MDA-MB-231, Buserelin inhibited cell proliferation, cell migration, and invasion (49, 144).

## GnRH-II Agonists

Few reports show the use of GnRHa-II against BC. In this case, [D-Lys6]-GnRHII (Table 1) induced the inhibition of cell migration in human BC cell lines HCC 70, MCF-7, MDA-MB-453, and T47-D (21).

## GnRH-I Antagonist

GnRH-ant do not induce an initial stimulation of the receptor; they compete for receptor occupancy, causing a rapid and reversible suppression of the hGnRHR-I response, reducing the onset time of therapeutic effects and also eliminating clinical “flare” (Figure 2). These two points comprise the major advantage of these molecules over the agonists. Initially, early GnRH-ant were developed by the replacement of aa residues in position 2 and position 3 due to the importance of these aa in receptor binding and functional effects (145). Later, substitutions of D-aa instead of L-aa in several positions, such as 2, 3, 6, and 10, further increased antagonistic activity. However, their clinical use was restricted by solubility limitations and anaphylactic reactions (146, 147). For these reasons, other modifications, such as the use of various substituents in the benzene ring of the D-Phe residue in position 2, the addition of groups such as Ac-D-2Nal, D-3Pal in the N-terminal part of the peptide, and the substitution by D-Ala in position 10 or different aa derivatives, mostly in positions 5, 6, and 8, improved the inhibitory activities of several antagonists (148).

### Cetrorelix

Cetrorelix is a GnRH-ant that has been shown as safe and effective in inhibiting the secretion of gonadotrophins (Table 2). Due to its capability to promote the suppression of LH and sex-steroid hormones, this GnRH-ant has been employed in the treatment of hormone-dependent cancers such as prostate and ovarian cancer (Figure 2) (149). Currently, the FDA indicated the use of this antagonist for inhibiting the premature LH surge in controlled ovarian hyperstimulation (COH) (150).

The direct antitumor effects of Cetrorelix in BC have been reported (Figure 2). In samples from patients diagnosed with TNBC, in cellular models such as TNBC cell lines (HCC1806 and HCC1937), estrogen-responsive tumor cell line MCF-7, and in xenografts carried out with the MCF-7 cell line in nude mice, the administration of Cetrorelix resulted in a significant decrease of cell proliferation (64, 67, 107). On the other hand, the antimetastatic activity associated with hGnRHR-I activation in breast tumors by the administration of Cetrorelix was confirmed. In this case, this antagonist reduced the formation of metastasis by the MDA-MB-231 cell line in a nude mouse model (63).

## GnRH-II Antagonists

Several works have revealed that other GnRH-ant-II exert a direct effect on malignant tumors (Figure 2). For example, Trptorelix-1 and SN09-2 (Table 2) induced cell death via the apoptotic process in cancer cells (72, 151). On the other hand, in cellular models of human BC such as MCF-7, MDA-MB-231, and T47-D, the administration of [Ac-D2Nal1, D-4Cpa2, D-3Pal3,6, Leu8, D-Ala10]-GnRHII (Table 2) induced the activation of apoptosis and promoted significant inhibition of tumor growth (74, 75).

## Non-peptide GnRH Antagonists and Their Use as Therapeutic Agents in BC

Several GnRHa peptides (Goserelin and Triptorelin) have been approved by their incorporation into the market. On the other hand, various GnRH-ant peptides, such as Cetrorelix, have been utilized clinically for several diseases, including in malignant tumors, such as BC. However, the clinical use of both types of GnRH analogs has been limited because they must be administered frequently by subcutaneous injection or sustained release formulations. For these reasons, several research groups have been developing non-peptide GnRH antagonists for clinical use (152). These are small non-peptidic molecules characterized by their oral administration and that exhibit better patient compliance during their clinical administration in comparison with injectable antagonists. They have titratable doses to accomplish the partial inhibition of sex-steroid hormones according to the disorder treated (Figure 2). They do not present, as do other GnRH-ant, the phenomenon of “flare,” and there is no risk of bone loss, such as that observed during long-term administration of GnRH agonists (78, 153).

Many classes of non-peptide GnRH antagonists have been described. They belong to different chemical families, such as Bicyclicpyrimidones/Pyrimidinediones, Uracils, Indoles, Quinolones, Quinolines and Phthalazines, Furan-2-Carboxamides, and Benzimidazoles; however, the majority of these have not achieved clinical application to date (154–157). The first non-peptidic compound evaluated in rat pituitary membranes was Ketoconazole (158, 159). Takeda Pharmaceutical developed the next non-peptide GnRH antagonist. This was called T-98475 and was described as the first highly potent and orally active non-peptidic GnRH antagonist (160). Later, a derivative from this molecule was synthesized, that is, the first non-peptide GnRH antagonist, which was evaluated in humans and is known as TAK-013 or Sufugolix (161). This compound was evaluated in a phase II study in patients with endometriosis, but it was not able to advance to phase III studies. Recently, a new compound was reported that is derived from TAK-013 and is denominated TAK-385 or Relugolix (162). This molecule was evaluated in a humanized mouse model, showing its ability to work as an antagonist of GnRH with the capacity to suppress, in a powerful, continuous, and reversible manner, the way that hGnRHR-I functions (Figure 2). Due the potent effect of TAK-385 as a non-peptide GnRH antagonist, it is currently employed in different clinical trials to determine its therapeutic use against several diseases including BC (163).

## Cytotoxic Analogs of GnRH Employed Against BC

Although the principal clinical effects of GnRH analogs have been associated with their ability to suppress estrogen action, as previously mentioned, in human clinical specimens of BC (49%) and in cell lines derived from malignant breast tumors, there are binding sites for hGnRH-I and hGnRH-II that could be successfully employed as therapeutic targets (Figure 2). With this in mind, molecules known as cytotoxic analogs have been designed. They are GnRH agonists conjugated to chemotherapeutic agents that combine hormonal and cytotoxic activity.

### Clinical Use of AN-152 and AN-207 Conjugates

AN-152 (also known as AEZS-108) and AN-207 are two cytotoxic analogs developed in 1996. In them, the GnRHa [D-Lys6]-GnRH is linked with Doxorubicin (DOX), a widely used anticancer agent. AN152 (AEZS-108) is conjugated to DOX and AN-207 is conjugated to 2-pyrrolino-DOX, a 500 ± 1,000 times more active derivative of DOX (164). Although the antitumor effect of both cytotoxic analogs as inhibitors of tumor growth has been demonstrated in an estrogen-independent and MXT mouse mammary cancer model (Figure 2), stronger effects of AN-207 as an inhibitor of tumor growth, as a promoter of the apoptotic index, and in terms of necrosis in tumors were also determined (165–167). On the other hand, the cellular targets of these conjugate molecules are specific for cancer cells that express hGnRHR-I, as demonstrated in MCF-7 (Figure 2). In this case, the expression of this receptor on cell surfaces promotes the conjugate-internalization process and metabolism, and evoked the cytotoxic effect of AN-152 (AEZS-108) (168). Similar results were observed in TNBC cell lines MDA-MB-231 and HCC-1806 (109, 110, 169, 170). In *in vivo* models, AN-207 gave rise to the regression of tumor growth in an MX-1 hormone-independent Doxorubicin-resistant human BC cell line (166, 171).

Emons et al. select AN-152 (AEZS-108) for a clinical trial because of its lower toxic adverse effects, and the authors also showed that the half-life of this compound in human serum is 2 h (172). Later, this same research group designed the first phase I study with 17 women who have a confirmed diagnosis of epithelial cancer of the ovary, endometrium, or breast. The AN-152 (AEZS-108) dose used in this trial was 267 mg/m^2^. This dose exhibited maximal tolerance without supportive medication and decrease in LH and FSH after administration of AESZ-108 (Figure 2) (173). Evaluation of AN152 (AEZS-108) in a phase II study and subsequent phase III clinical trials (https://clinicaltrials.gov/ct2/show/NCT01767155) was performed in patients with ovarian cancer and in patients with endometrial cancer, confirming in both of these the anticancer activity of this cytotoxic analog (174).

### Cytotoxic Analogs of GnRH That Employ Specific Spacers/Linkers Such as Oxime Bond and Hydrazone and Their Evaluation Against BC

Other conjugated anticancer GnRH agonists have been designed. In order to increase stability in the human serum of these molecules, GnRH peptides were attached to anticancer drugs through several spacers/linkers, such as oxime bond and hydrazone bond (175, 176). Daunorubicin was conjugated via oxime bond to GnRH-III; this modification increased the stability of the GnRH bioconjugates in human serum at least for 24 h and did not affect its antitumor properties in the MCF-7 cell line (175). Incorporation of anticancer drugs such as the anthracyclines, Daunorubicin and Doxorubicin, to GnRH-III and [D-Lys6]-GnRH analogs through oxime, hydrazine, or ester bonds as spacers showed potent anticancer action in the MCF-7 cell line and increased their half-life in human serum (176). New cytotoxic compounds include two GnRH analogs ([D-Lys6] GnRH-I and [D-Lys6] GnRH-II), with a link between them and the chemotherapeutic agent, Daunorubicin (Dau), through an enzyme-labile spacer using oxime bond. In this work, it was shown that both conjugates possess similar receptor-binding affinity and antitumoral properties, such as an apoptotic effect in MCF-7 cells and in the colon cell line, HT-29 (Figure 2) (62). Five conjugates of Paclitaxel (PTX) were linked to the GnRH antagonist Degarelix as targeting moiety and were evaluated in MCF-7 and HT-29 cell lines as anticancer agent. The half-lives of all of the conjugates in human serum were close to 10 h, and high-affinity GnRH-receptor binding and an antiproliferative effect on MCF-7 cells were shown (177).

## Conclusions

All of the information reported in the present review supports the use of hGnRH type I and hGnRH type II analogs, non-peptide GnRH antagonists, and the cytotoxic analog complex to GnRH as adjuvants in the therapy against BC. However, it is important to consider that, for the use of GnRH analogs in clinical practice, it is necessary to have an understanding of the actions of these molecules in breast tumors, such as the pharmacology of agonists on the breast tumor, defining in detail their potency, and the signaling pathways involved, as well as possible adverse effects, among others. Similarly, extrapolation of the data obtained through studies carried out in animal models or in cell lines must be performed with caution. Even so, many clinical studies have shown that hGnRH agonists have the ability to reduce BC growth and disease recurrence, principally in patients with TNBC. In addition, these agonists are able to reduce tumor migration, including in advanced cases. Another important point to consider is that GnRHa might exert systemic and local action on BC. The use of GnRH analogs as adjuvant therapy against BC has demonstrated their ability to reduce circulating concentrations of estrogen, with the concomitant reduction of tumor growth. Likewise, agonists might exert a local effect by direct activation of the GnRHR-I and the concomitant reduction in tumor growth and prevention of distant metastasis. This dual effect could improve the clinical response, principally in combined therapies. Finally, although the use of GnRH analogs, non-peptide GnRH antagonists, and cytotoxic analogs of GnRH requires more clinical trials for their clinical implementation, the hGnRH/hGnRHR system comprises a very promising target for further pharmaceutical development in the treatment of BC, especially for the treatment of advanced stages of this disease.

## Author Contributions

AA-R and MH-R conceptualized and wrote the first and final versions of the article. NG and J-CO-M contributed to refining the article. GM-N improved the GnRH and GnRH receptor section. MP-S and EL-M wrote and improved the clinical section. All of the authors read and approved the final version of the article.

### Conflict of Interest

The authors declare that the research was conducted in the absence of any commercial or financial relationships that could be construed as a potential conflict of interest.
